# Regulatory RNA Ern0160 controls *Enterococcus faecium* virulence through direct modulation of expression of LysM domain-containing proteins

**DOI:** 10.1186/s12864-025-12464-2

**Published:** 2026-01-05

**Authors:** Loren Dejoies, Valérie Bordeau, Killian Le Neindre, Sophie Reissier, Kevin Arnould, Brice Felden, Svetlana Chabelskaya, François Guérin, Charlotte Michaux, Vincent Cattoir

**Affiliations:** 1 Department of Clinical Microbiology, Rennes University Hospital, Rennes, France; 2 University of Rennes, Inserm UMR_S 1230, Bacterial RNAs and Medicine, Rennes, France; 3 National Reference Center for Antimicrobial Resistance (Lab Enterococci), Rennes, France

**Keywords:** *E. faecium*, sRNA, Regulation, Virulence

## Abstract

**Background:**

*Enterococcus faecium* is a commensal of the human gut microbiota that can become an opportunistic pathogen, particularly in immunocompromised individuals. Small RNAs (sRNA) are thought to contribute to this shift by enabling rapid bacterial adaptation to environmental changes. Despite this, knowledge of sRNA in *E. faecium* remains limited. Ern0160, in particular, has attracted interest for its involvement in antibiotic and biocide responses, as well as its role in intestinal colonization in a murine model.

**Results:**

In this study, we investigated the functions of Ern0160 in *E. faecium* Aus0004 reference strain and sought to identify its mRNA targets. Transcriptomic and in silico analyses revealed potential regulatory targets, including two homologous genes encoding LysM-containing domain proteins (EFAU004_01059 and EFAU004_01150), both associated with enterococcal pathogenicity. Experimental validation confirmed that increased expression of Ern0160 led to repression of these genes. We further demonstrated direct and specific interactions between Ern0160 and the two homologous target mRNAs. Functional assays in the *Galleria mellonella* larvae infection model showed that deletion of Ern0160 resulted in increased host mortality, whereas deletion of its targets genes resulted in decreased mortality. These results are consistent with previous findings linking these genes to *E. faecium* virulence in murine model of systemic and urinary tract infections.

**Conclusions:**

Our findings suggest that Ern0160 contributes to a regulatory network that modulates *E. faecium* colonization and infection by targeting genes involved in antimicrobial response and virulence. This study highlights the potential of regulatory RNAs such as Ern0160 to shape the pathogenic behavior of a multi-drug resistant and clinically significant bacterium.

**Supplementary Information:**

The online version contains supplementary material available at 10.1186/s12864-025-12464-2.

## Background

*Enterococcus* spp. are commensal bacteria that reside in the human gastrointestinal tract, representing less than 1% of the gut microbiota in healthy adult [1]. Although generally benign, enterococci can become major opportunistic pathogens, causing various infections (e.g. urinary tract and intra-abdominal infections, bacteremia, endocarditis), particularly in immunocompromised patients admitted to intensive care units and receiving long-term antimicrobial treatments [2, 3]. Among the different species, *Enterococcus faecalis* and *Enterococcus faecium *are the primary culprits responsible for hospital-acquired infections worldwide. *E. faecium* alone accounts for 15–25% of enterococcal infections. This species presents a particular challenge because of resistance to multiple antimicrobials, including penicillins, aminoglycosides (high-level), and glycopeptides – resulting in vancomycin-resistant enterococci (VRE) – which complicates treatment efforts [4].

Recent Whole Genome Sequencing (WGS) studies have revealed distinct lineages within *E. faecium*: the hospital-associated (HA) lineage (clade A) and the community-associated (CA) lineage (clade B). The HA lineage, in particular, displays a greater diversity in the accessory genome, which contributes to its persistence in antimicrobial-rich environments of hospitals [5]. These features position *E. faecium* as one of the most concerning pathogens affecting human health. However, in comparison to *E. faecalis*, *E. faecium* possesses a more limited set of virulence factors. Only a few determinants have been linked to pathogenicity in animal models, such as the Acm adhesion protein [6, 7], the Esp cell surface protein [8], the *ebpABC*_*fm*_ pili gene cluster [9] and the BepA phosphotransferase system permease [10].

Recently, small RNA (sRNA) regulators have emerged as novel virulence factors, originating from intergenic regions once thought to be non-functional. These sRNAs form a new class of regulatory elements that operate at the post-transcriptional level [11]. These bacterial regulators influence messenger RNA (mRNA) targets either independently or with the assistance of protein partners such as Hfq or ProQ [12]. While extensively investigated in Gram-negative bacteria, evidence for analogous global RNA-binding proteins with comparable post-transcriptional roles in Gram-positive bacteria remains scarce [13], suggesting distinct mechanisms of post-transcriptional regulation between Gram-positive and negative bacteria.

As the understanding of post-transcriptional regulation continues to expand, growing evidence emphasizes a shared trait across both Gram-positive and -negative bacteria: sRNAs play a critical role in dynamic adaptive responses, including virulence and antimicrobial resistance. Despite extensive documentation of sRNA functions in bacteria such as *Escherichia coli*, *Salmonella enterica* serovar Typhimurium, *Staphylococcus aureus*, and *E. faecalis*, functional insights into sRNAs in *E. faecium* remain limited.

In 2017, the first sRNome expressed from non-annotated regions of the reference strain Aus0004 was described under sub-inhibitory daptomycin concentrations [14]. More recently, transcription start site (TSS) mapping under physiological non-stress conditions (rich media) has expanded the predicted sRNA repertoire in *E. faecium* to approximately 165 candidates [15]. Among these, 17.5% (29 out of 165) have been experimentally validated through Northern blot analyses [14, 15], although their biological functions remain uncharacterized. Subsequent studies on *E. faecium* Aus0004 have focused on sRNA_0030 and sRNA_0160, now designated as Ern0030 and Ern0160, confirming sRNA-mediated regulation of virulence and antibiotic resistance [11, 16].

Initial characterization of Ern0160 by Sinel et al. reported a predicted length of 362 to 370 base pairs. The study demonstrated Ern0160 accumulation during bacterial growth via Northern blot and observed altered expression under daptomycin stress, supported by RNA-seq and quantitative reverse transcription PCR (RT-qPCR). Furthermore, Ern0160 was identified as a conserved element within both HA and CA lineages, forming part of the core genome [14]. More recent findings have linked Ern0160 to the bacterial stress response to biocides (chlorhexidine digluconate, benzalkonium chloride, and PVP-iodine) [17], as well as to intestinal colonization in a murine model [11].

Here, we aimed to further unravel Ern0160 functions and identify its specific mRNA targets. Through RNA-seq analysis, two target genes were identified, supporting the involvement of Ern0160 in antibacterial activity regulation. Follow-up analyses confirmed conclusive evidence of the direct interaction between Ern0160 and both EFAU004_01059 and EFAU004_01150, highlighting the regulatory impact of this sRNA on its targets. Finally, using the *Galleria mellonella* infection model, we assessed the importance of Ern0160 and its targets in virulence regulation. The absence of this sRNA led to increased caterpillar mortality. Overall, this study provided new insights into the post-transcriptional regulatory functions of Ern0160, with implications for antibacterial responses and virulence in *E. faecium*.

## Methods

### Bacterial strains and growth conditions

All bacterial strains and plasmids used in this study are listed in Supplementary Table S1. All primers are listed in Supplementary Table S2. *E. faecium* Aus0004 was selected as the reference strain as it belongs to the hospitalized-associated (HA) clade A [18], and its genome has been fully sequenced and annotated (GenBank accession no. CP003351) [19]. Three strains were constructed by Reissier et al. [11]. and are referred to in the manuscript as follows: the *ern0160*-deleted strain (Δ0160), the *ern0160*-deleted strain carrying an empty promotor-less pAT29 vector (Δ0160_pAT29), and the *ern0160*-overexpressing strain (Δ0160_Ern0160). An additional strain overexpressing the *ern0160mut13* variant, referred to as Δ0160_Ern0160mut13, was constructed using the same approach in this study. Briefly, regions upstream (P1) and downstream (P2) of the binding site in Ern0160 were amplified using P1 and P2 primer pairs (Table S2). The resulting fragments were assembled into the pAT29 shuttle vector using the NEBuilder HiFi Assembly Kit (NEB), yielding the recombinant plasmid pAT29Ωern0160mut13. This plasmid was first transformed into *E. coli* DH5α competent cells. After plasmid extraction, it was introduced into the Δ0160 mutant strain by electroporation. The Ern0160 targets gene deletion mutants - Δ*efau004_01059*; Δ*efau004_01150* and the double mutant Δ*efau004_01059_01150 -* were kindly provided by the Hartke group [20]. All *E. faecium* strains were grown at 37 °C under standard conditions in brain-heart infusion (BHI; Oxoid) broth and/or on BHI agar (Oxoid). When required, media were supplemented with 300 mg/L of spectinomycin.

### RNA isolation and sequencing

Total RNA was isolated from cultures at the late exponential (LE) growth phase using the Quick-RNA Miniprep kit (Zymo). Residual chromosomal DNA was removed by treating RNA samples with the Turbo DNA-free kit (Life Technologies).

For RNA sequencing, two independent biological replicates were prepared for *E. faecium* Aus0004 and each mutant strain, all harvested during the LE growth phase. Sequencing was outsourced to ViroScan3D (Lyon, France, www.viroscan3d.com). RNA quantification and quality assessment were performed using a 2100 Bioanalyzer (Agilent Technologies). Strand-specific libraries were prepared using the Ovation Universal Prokaryotic RNA-Seq kit (Nugen) with custom rRNA depletion designed for *E. faecium* (AnyDeplete). Sequencing was performed on the Illumina NextSeq500 platform using a multiplexed protocol with single-end 75-bp reads.

Resulting reads were mapped to the *E. faecium* Aus0004 genome using CLC Genomics Workbench software v10.0.1 (Qiagen). Gene expression levels were quantified, and differential expression analysis was conducted using the DESeq2 package in R [21]. These genes were further analyzed using Gene Ontology (GO) enrichment analysis. IDs were obtained from UniProt and submitted to the DAVID Bioinformatics module (NCBI). Functional annotation categories were plotted based on their significance (*P*-value < 0.05). Raw and processed RNA-seq data have been deposited in the Gene Expression Omnibus (GEO) repository at the National Center for Biotechnology Information (NCBI) under accession number GSE239910.

### In silico predictions, in vitro transcription, RNA labeling, and gel-shift assays

IntaRNA (v.3.3.1) was used to predict base-pairing interactions between Ern0160 and putative mRNA targets [22]. In vitro transcription of selected candidates was performed using PCR-generated DNA amplicon and the MEGAscript T7 kit (Ambion), following the manufacturer’s instructions. Transcription templates were amplified from *E. faecium* Aus0004 genomic DNA (extracted with InstaGene™ Matrix, BioRad) using forward primers containing T7 promoter sequences (detailed in Supplementary Table S2). Transcribed RNAs were 5’ end-labeled with [γ^32^-P]^−^ATP (Amersham Biosciences) using T4 polynucleotide kinase (Invitrogen). Labeled and unlabeled RNAs were purified on 6% acrylamide-urea gels, eluted in elution buffer (20 mM Tris-HCl pH 7.5, 250 mM NaCl, 1 mM EDTA, 1% SDS) at 37 °C, followed by ethanol precipitation. RNA concentrations were determined using a Qubit fluorometer (Thermo Fisher Scientific), and samples were stored at -80 °C.

Electrophoretic mobility shift assays (EMSAs) were carried out as previously described [23]. Briefly, transcripts were denatured in 50 mM Tris/HEPES (pH 7-7.5) and 50 mM NaCl for 2 min at 80 °C. Refolding was performed for 10 min at 25 °C by adding MgCl_2_ to a final concentration of 5 mM, followed by cooling on ice. Binding reactions were assembled by incubating 0.25 to 0.5 pmol of labeled Ern0160 with increasing molar excesses of target mRNAs in binding buffer (50 mM Tris-HCl (pH 7.5), 50 mM NaCl, and 5 mM MgCl_2_) at 25 °C for 20 min. For specificity validation, 25 pmol of unlabeled Ern0160 was added. Reactions were mixed with 10% glycerol and loaded onto native 5% polyacrylamide gel containing 5% glycerol. Gels were visualized using a Typhoon FLA 9500 scanner (GE Healthcare) or by autoradiography. Specificity of interaction was further assessed by introducing deletions in the predicted binding regions of the mRNA targets, generating modified transcripts referred to as ‘mRNA’*mut*.

### qRT-PCR assays

To quantify expression levels of selected mRNA targets, one-step quantitative reverse transcription PCR (qRT-PCR) was conducted using the Power SYBR^®^ Green RNA-to-CT™ 1-Step kit (Thermo Fisher Scientific), following the manufacturer’s instructions. Primers used for amplification are listed in Supplementary Table S2. Transcript levels were obtained using the ΔΔCt method and the gene expression levels were normalized towards the house keeping gene *adk*. Prior to this, primer efficiencies were measured, with 99% efficiency for *adk* and 103% for *lysM*. For statistical analysis, a two-tailed Mann-Whitney test was applied. Data analysis and visualization were carried out using GraphPad Prism 7 (GraphPad Software, Inc.).

### Infection of *Galleria mellonella* larvae with *E. faecium*

The *Galleria mellonella* infection procedure with *E. faecium* was performed following the protocol previously described by Lebreton et al. [24]. Briefly, larvae weighing approximately 300 mg and measuring around 3 cm in length were subcutaneously injected using a syringe pump (KD Scientific, Holliston, MA) with washed *E. faecium* cells harvested from an overnight culture in BHI medium. Each larva received 10 µL of sterile saline buffer containing 5 × 10⁷ (± 0.5) CFU. For each experimental group, 10 larvae were infected, and all experiments were conducted in triplicate. Larval survival was monitored daily over a five-day period post-infection, and mortality rates were recorded accordingly.

### Statistical analysis

For RNA-seq data, false discovery rates (FDR) were calculated using the Benjamini-Hochberg method. For in silico predictions using IntaRNA, the top ten targets were selected based on the minimum free energy (ΔG) of hybridization and accessibility of the target region. For RT-qPCR experiments, differences between two strains were evaluated using Student’s t-test. EMSA binding differences across multiple mRNA concentrations were assessed using Welch’s *t*-test to account for unequal numbers of replicates (four replicates for conditions 1–5 and three replicates for conditions 6 and 7). For *G. mellonella* survival experiments, comparisons between two strains were performed using the Mantel-Cox (log-rank) test.

## Results

### Genetic environment of Ern0160 in Aus0004 strain and conservation

**Fig. 1:**
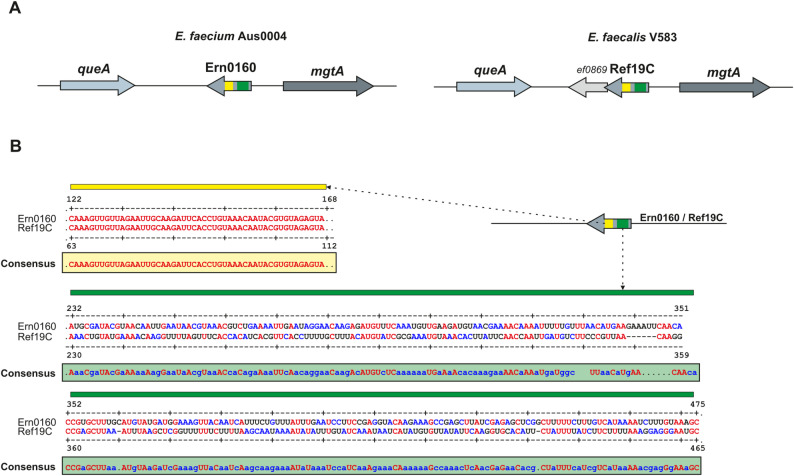
Conservation analysis. **A** Genomic comparison showing the conserved context of Ern0160 in *E. faecium* Aus0004 and Ref19C in *E. faecalis* V583. **B** Nucleotide alignment of Ern0160 and Ref19C with the derived consensus sequence. Yellow and green blocks indicate regions of sequence homology

According to the genetic coordinates reported by Sinel et al. [14]. , the sRNA Ern0160 is located on the negative strand of the *E. faecium* Aus0004 genome, between *efau004_00234*, which encodes an S-adenosylmethionine-tRNA ribosyltransferase-isomerase (*queA*), and *efau004_00235*, which encodes a calcium-translocating P-type ATPase (*mgtA*) (Fig. 1A). No open reading frame (ORF) has been identified within the Ern0160 sequence. Interestingly, Ern0160 shared 24% nucleotide identity with the sRNA Ref19C of *E. faecalis* [25], corresponding to a 113-nucleotide region located near the center of the Ref19C sequence (Fig. 1A and B). As shown, two regions appear homologous: one highly conserved (yellow) and one less conserved (green). The genetic context surrounding Ref19C in *E. faecalis* closely mirrors that of Ern0160 in *E. faecium*: *ef0868*, homologous to *efau004_00234*, encodes for *queA*, and *ef0871*, homologous to *efau004_00234*, encoding for *mgtA*. It is noteworthy that no homologous counterpart of the *E. faecalis* gene *ef0869* has been identified in *E. faecium* (Fig. 1).

### Global transcriptomic analysis

**Fig. 2:**
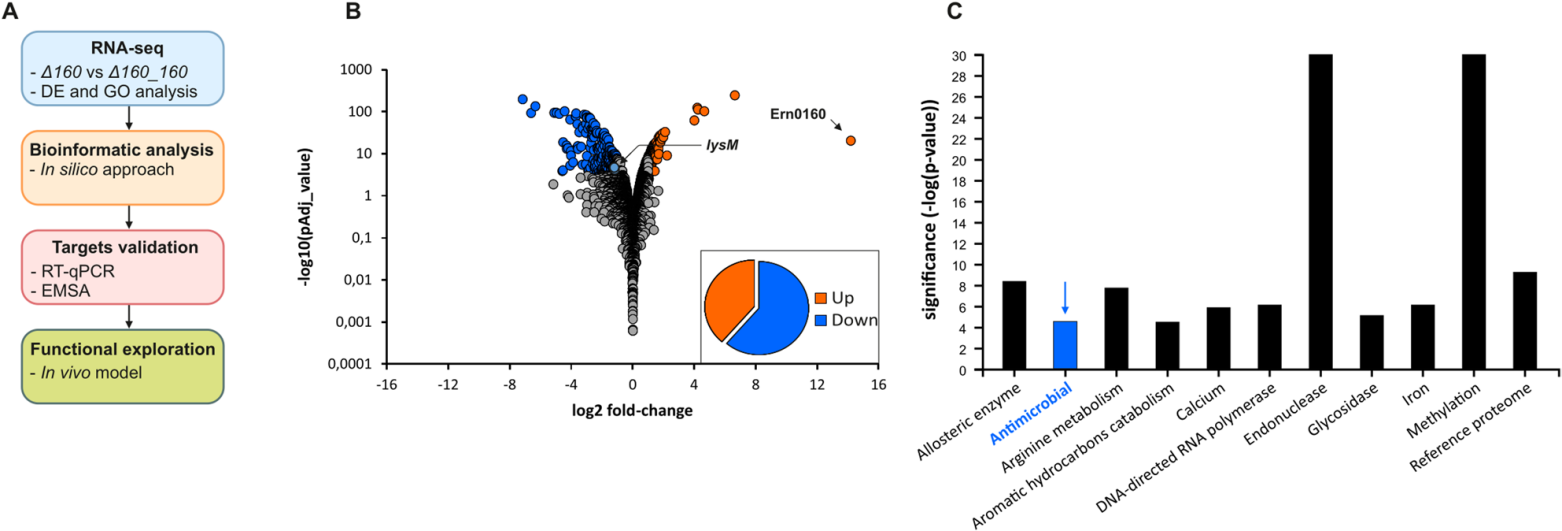
RNA-seq analysis. **A** Schematic of the strategy used to identify direct targets of Ern0160. **B** Volcano plot showing differential gene expression in Δ0160_Ern0160 vs. Δ0160_pAT29. Blue: significantly downregulated transcripts; Orange: significantly upregulated; grey: not significant (criteria: Padj < 0.001, |Log2FC| > 1). **C** Summary of Gene Ontology (GO) enrichment analysis for differentially expressed transcripts

To identify direct targets of Ern0160, we implemented a comprehensive strategy combining diverse systematic and unbiased approaches, as outlined in Fig. 2A. We started with a global transcriptomic approach using RNA-seq to compare the transcriptomes of the *E. faecium* Aus0004 strain (control) with the Δ0160 mutant and separately, the Δ0160_pAT29 strain with the Ern0160-overexpressing strain (Δ0160_Ern0160). Each RNA-seq library yielded approximately 20 million reads, with 72% to 91% of the reads mapping to the *E. faecium* Aus0004 genome. This resulted in average genome coverage ranging from 326× to 700× (Supplementary Table S3). Experimental duplicates displayed strong reproducibility across conditions, with Pearson correlation coefficients (R²) exceeding 0.92 (Supplementary Figure S1). The average read count per coding sequence (CDS) ranged from 3,411 to 7,233 (Supplementary Table S3).

Differential expression analysis using DESeq2 identified only 13 genes with statistically significant transcriptional changes between the WT and Δ0160 strains. These genes met the thresholds of a log_2_ fold change (FC) < -1 or > 1 and an adjusted P-value < 0.001 (Supplementary Table S4, Supplementary Figure S2). In contrast, comparison between Δ0160_Ern0160 and Δ0160_pAT29 strains revealed a substantial deregulation of 419 genes. Among these, more than 60% (257 genes) showed reduced expression upon Ern0160 overexpression (Supplementary Table S4; Fig. 2B).

It is notable that deletion of Ern0160 led to only minimal gene expression changes, whereas overexpression triggered widespread transcriptional alterations. No genes were found to be commonly regulated in both comparisons, limiting the utility of cross-comparison. This discrepancy may be explained by the mode of action of sRNAs, which typically inhibit translation of their targets. Overexpression in rich medium thus provides a useful proxy to mimic physiologically relevant induction states and enables detection of regulatory interactions that may remain silent under basal laboratory conditions. This rationale aligns with common practice in the sRNA field, where overexpression is routinely used to reveal direct regulatory effects and is generally more likely to elicit observable changes than deletion.

Focusing on the differentially expressed genes in the Ern0160-overexpressing strain, Gene Ontology (GO) enrichment analysis using DAVID Bioinformatics database revealed several enriched gene categories, including a set associated with antimicrobial pathway (Fig. 2C). Since the RNA-seq data provide information on both direct and undirect targets of Ern0160, encompassing several functional categories of interest, we decided to use in silico base pairing prediction approaches and cross-compare the predicted targets with our RNA-seq data to discriminate between direct and indirect targets.

### Direct mRNA-sRNA in silico predictions

Interestingly, the two top mRNA target candidates identified through in silico analysis using IntaRNA corresponded to the two genes belonging to the enriched antimicrobial pathway identified in the transcriptomic data, both of which were downregulated in the Ern0160 overexpression strain.

**Fig. 3:**
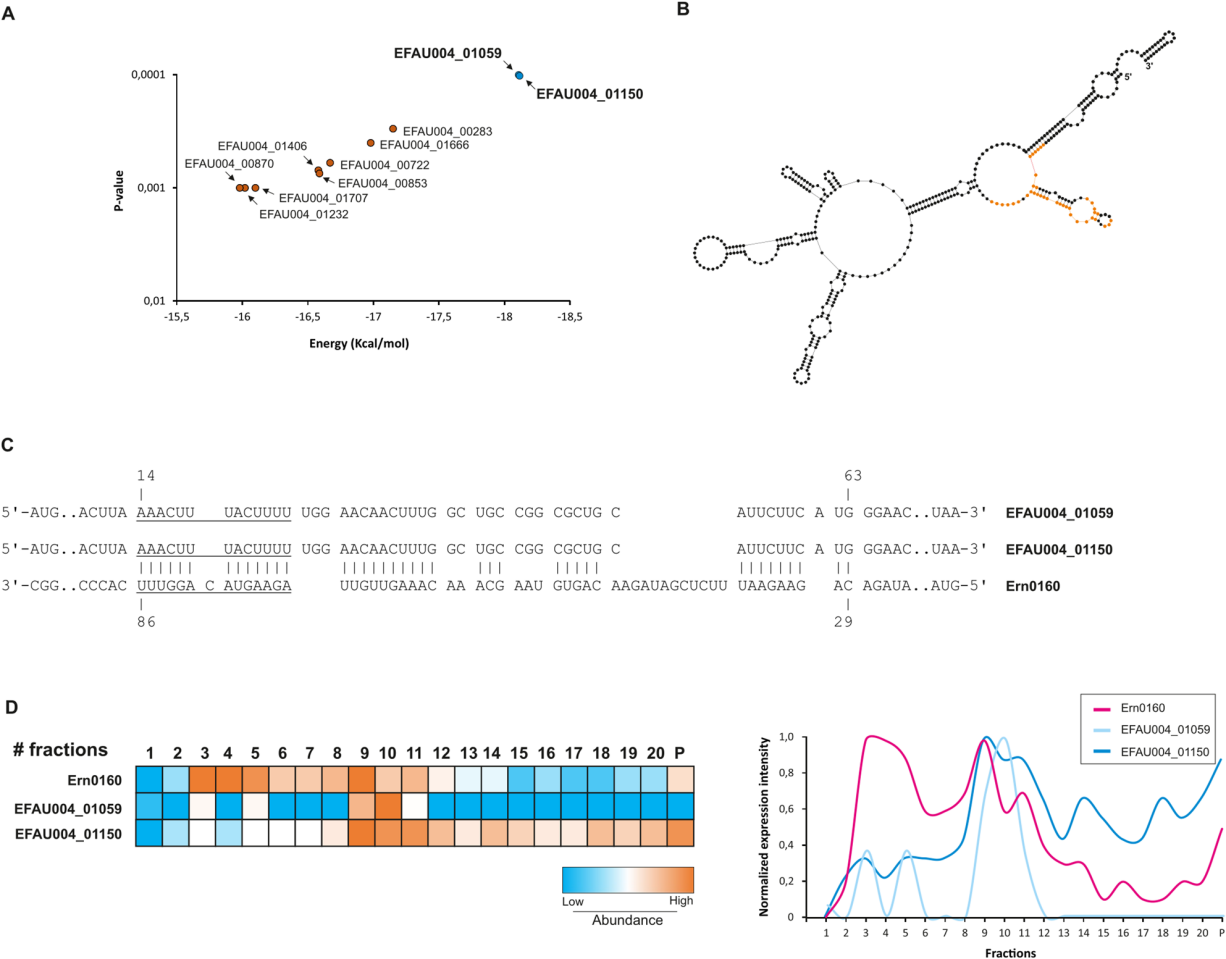
Target predictions. **A** Top ten mRNA targets predicted using IntaRNA [22], with the two most promising targets highlighted in blue. **B** Predicted secondary structure of Ern0160 using RNAstructure [28]. Nucleotides involved in base-pairing interactions are shown in orange. **C** In silico-predicted base-pairing interactions between Ern0160 and *efau004_01059/01150* mRNAs. The interaction zone (underlined) was deleted in subsequent experiments (see Fig. 4). **D** Sedimentation profiles from Grad-seq showing distribution of Ern0160, *efau004_01059*, and *efau004_01150* across glycerol gradient fractions. RNA abundance across fractions is quantified, with orange indicating the fractions with the highest abundance and blue indicating the fractions with the lowest abundance ; “P” denotes the pellet fraction

These genes, *efau004_01059* and *efau004_01150* (Fig. 3A) encode for LysM domain-containing proteins - a widespread conserved class of bacterial proteins known for their ability to bind peptidoglycan, a fundamental component of the bacterial cell walls [26]. These proteins have been implicated in diverse bacterial processes, including cell wall remodeling, pathogenesis, and host-microbe interactions, including in *E. faecium* [20, 27]. 

Sequences analysis revealed a striking 97.6% nucleotide identity between the two genes, with *efau004_01059* containing an additional 15 nucleotides near located near its middle. Remarkably, the predicted base-pairing region with Ern0160 was completely conserved in both genes and located within their respective open reading frames (ORF) (Fig. 3B and C). Recently, a Grad-seq experiment conducted in *E. faecium* Aus0004 offered further insight into potential Ern0160-mRNA interactions [29]. This comprehensive method separated cellular complexes via glycerol gradient centrifugation, followed by RNA-seq and mass spectrometry to infer interactions based on co-migration profiles. Comparison of gradient distributions showed Ern0160 localizing initially in low molecular weight (LMW) fractions (fractions 3 to 5), indicative of its free form. Strikingly, Ern0160 also peaked in higher molecular weight (HMW) fractions (fractions 8 to 10), suggesting its incorporation into a larger complex. Both LysM mRNA targets were detected in fractions 9 to 11, consistent with possible interactions with Ern0160 (Fig. 3D). We further explored the dataset for candidate RNA-binding proteins that could potentially interact with Ern0160. Well-characterized regulators such as S1, Cold-shock proteins, and Khp proteins were examined. However, none exhibited a distribution pattern clearly matching that of Ern0160, preventing any direct assignment of a protein partner.

### Experimental investigations of selected mRNA target candidates

We experimentally validated selected mRNA targets using two complementary approaches: electrophoretic mobility shift assays (EMSA) to assess direct sRNA-mRNA interactions, and qRT-PCR to evaluate the impact of Ern0160 on target transcript levels.

**Fig. 4:**
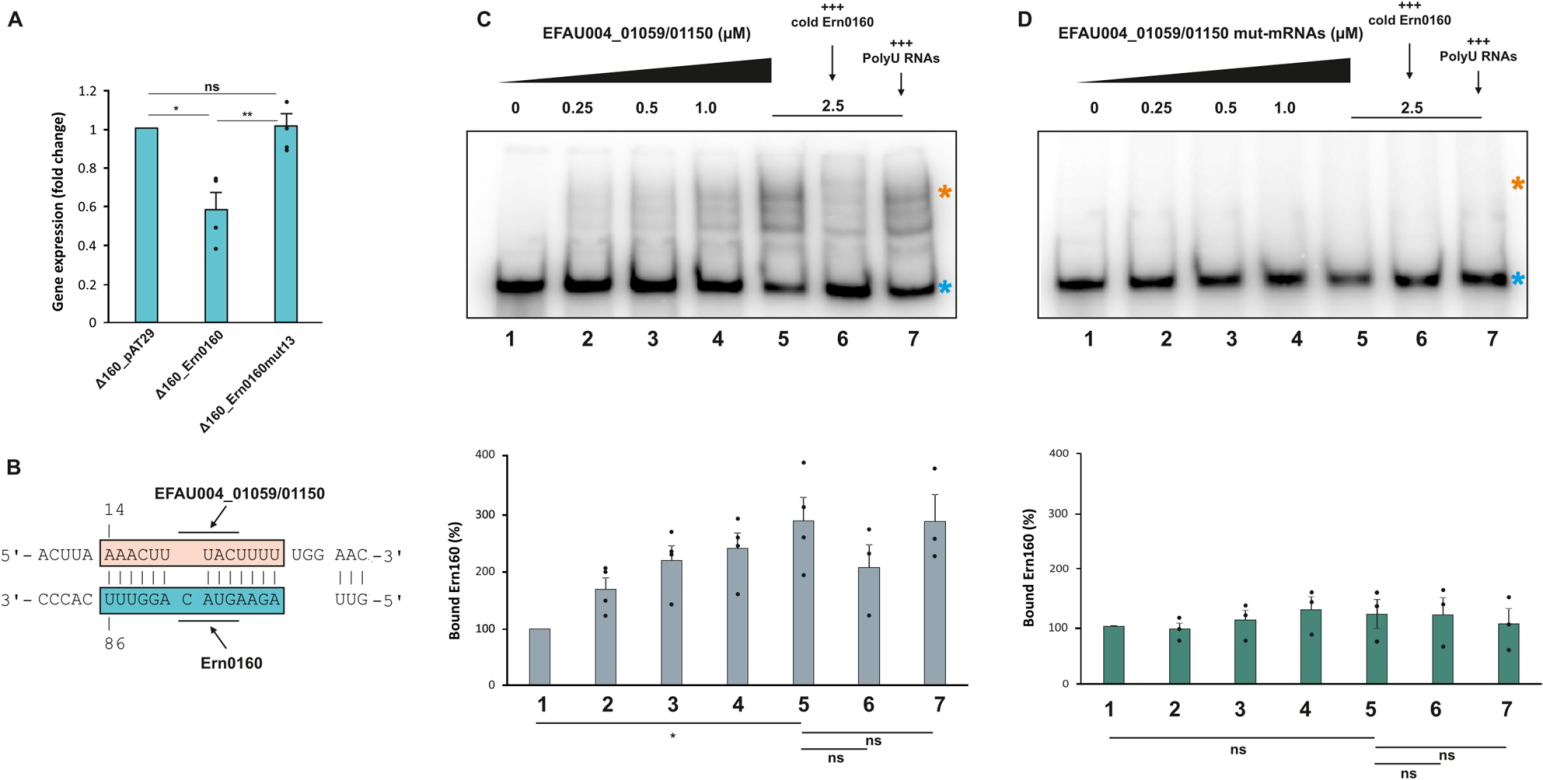
Direct interactions. **A** qRT-PCR quantification of *efau004_01059/01150* mRNA levels during late exponential phase under different Ern0160 conditions: absence, overexpression of wild-type Ern0160, and overexpression of the Ern0160mut13 variant. Error bars represent standard deviation. Statistical significance: ns (not significant), **P < 0.01 (Δ160_pAT29 versus Δ160_Ern0160 : 0.0007 and Δ160_Ern0160 versus Δ160_Ern0160mut13 : 0.0002) (Student’s t-test). **B** Schematic representation of the 13-nucleotide deletion within the predicted interaction zone between the *lysM* mRNA target and the Ern0160 sRNA. The blue box indicates the region deleted in the Ern0160 sRNA (panel A), whereas the salmon box marks the sequence removed from the synthetic *lysM* mRNA target (panel **D**). **C** EMSA: Upper panel—shift assay using labeled Ern0160 with increasing concentrations of target mRNAs (0.25, 0.5, 1.0, 2.5 µM; lanes 1–5). Lane 6: competition with cold Ern0160; lane 7: control with polyU. Lower panel—Quantification of complex formation (bound Ern0160) from three biological replicates was performed using ImageJ software. **D** EMSA using the mutant target RNA (*efau004_01059/01150mut*): same layout as (**C**). Absence of complex formation confirms specificity of the interaction. In both (**C**) and (**D**): blue asterisks indicate free labeled Ern0160; orange asterisks indicate sRNA*–mRNA* complexes. Error bars: standard deviation; statistical significance: ns (not significant), *P < 0.05 (0.0199) (Welch t-test). The full-length uncropped EMSA gels corresponding to panels **C** and **D** are provided in Supplementary Figure S3

We first examined the effect of Ern0160 on the expression level of its putative targets *efau004_01059* and *efau0004_01150*, using qRT-PCR. Primers were designed to amplify both genes simultaneously (Supplementary Table S2). Our results showed a significant downregulation of *efau004_01059/1150* expression upon strong overexpression of Ern0160 (Fig. 4A). However, overexpression of a truncated Ern0160 variant lacking the 13-nucleotide pairing region (Fig. 4B; blue box) failed to repress *efau004_01059/01150* expression, restoring transcript levels to those observed in the Δ0160 strain (Fig. 4A).

To test the direct interaction between Ern0160 and its predicted targets, we performed in vitro EMSA assays using synthetic RNAs. One RNA contained the complete predicted base-pairing region (Fig. 4C; Supplementary Table S2), while the other carried a 13-nucleotide deletion within the 40-nucleotide interaction zone (Fig. 4B, salmon box; Fig. 4D). Gel shift assays revealed the formation of a dynamic complex between Ern0160 and *efau004_1059/1150* transcripts (Fig. 4C). Quantification using ImageJ showed that the proportion of complexed labelled Ern0160 increased with higher mRNA concentrations. The addition of cold (unlabelled) Ern0160 appeared to disrupt complex formation; although the quantification did not yield a significant result, the trend is still consistent with binding specificity. In contrast, polyU had no effect (Fig. 4C). Using a synthetic RNA variant, *efau004_01059/1150mut*, carrying a 13-nucleotide deletion within the predicted base-pairing region, we further validated the specificity of the interaction. This truncated mRNA failed to form a complex with Ern0160, confirming that the interaction is sequence-specific (Fig. 4D).

### Animal infection

**Fig. 5:**
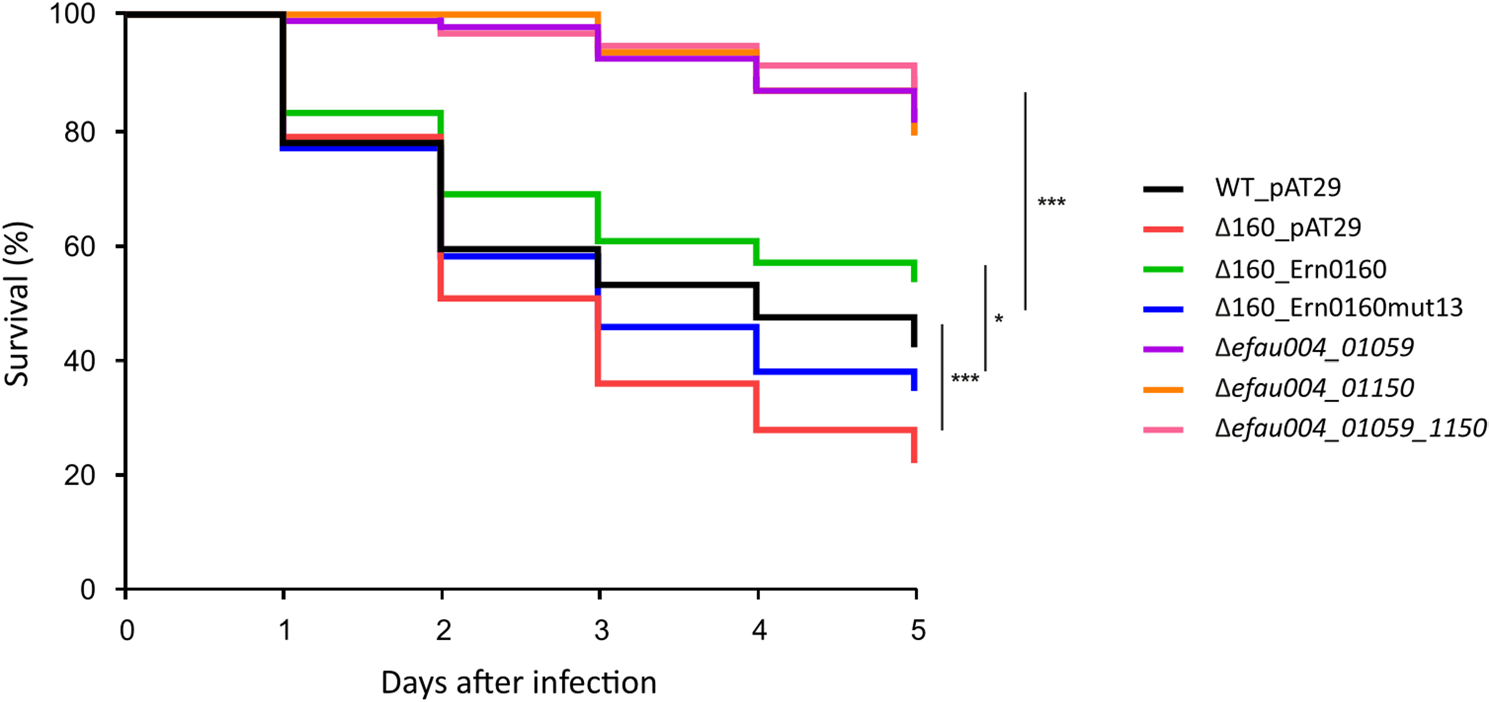
*Ern0160* affects virulence. Survival curves of *G. mellonella* larvae over five days following infection with various *E. faecium* strains: wild-type (black), Δ0160_pAT29 (red), Δ0160_Ern0160 (green), Δ0160_Ern0160mut13 (blue), Δ*efau004_01059* (purple), Δ*efau004_01150* (orange), and Δ*efau004_01059_01150* (pink). Data represent mean ± standard deviation from independent biological replicates. Statistical significance: **P < 0.01, ***P < 0.001 (WT_pAT29 versus Δ160_pAT29: 0.0001; Δ160_pAT29 versus Δ160_Ern0160 : 0.000089; Δ160_Ern0160 versus Δ160_Ern0160mut13: 0.003) (Mantel-Cox (log-rank) test)

Building on previous findings that highlighted the role of Ern0160 in intestinal colonization [11], and considering that both LysM targets have been implicated in virulence [20], we hypothesized that the deletion or overexpression of Ern0160 might influence the virulence phenotype. To test this, we used the established *Galleria mellonella* infection model and monitored larval survival following infection with the WT, the Δ0160_pAT29, the Δ0160_Ern0160 and the Δ0160_Ern0160mut13 strains. As shown in Fig. 5, larvae infected with the Δ0160 strain exhibited significantly reduced survival compared to those infected with the WT strain, indicating increased virulence. In contrast, overexpression of Ern0160 was associated with a trend toward improved larval survival, while overexpression of the Ern0160mut13 variant (lacking the base-pairing region) showed the opposite trend. As previously reported [20], both the single and double LysM target mutants were associated with increased larval survival. Taken together, these results suggest that in the absence of Ern0160, its target genes are no longer properly regulated, leading to increased expression of factors that enhance virulence in vivo.

## Discussion

Commensal bacteria such as *E. faecium* often navigate a fine line between colonization and pathogenicity. A key strategy in this balance is the modulation of virulence and/or antibiotic resistance, influenced by environmental cues within the host [30]. Our previous studies have shown that the expression of the small RNA (sRNA) Ern0160 is responsive to stressors commonly encountered in the gastrointestinal tract, including antibiotics such as daptomycin and ciprofloxacin, as well as environmental pressures like biocides [17, 31]. These findings suggest Ern0160 could act as a molecular sensor, integrating host-derived signals to modulate gene expression for optimal fitness in fluctuating environments.

In this study, we aimed to systematically identify the targets of Ern0160 using an integrative strategy combining global transcriptomic profiling, in silico interaction prediction, and functional phenotypic validation (Fig. 2). Through this multi-layered approach, we identified two high-confidence mRNA targets—*efau004_01059* and *efau004_01150*—which encode LysM domain-containing proteins. The high sequence similarity between the two genes, including complete identity in the predicted Ern0160 interaction region, suggests they may function redundantly in the bacterial cell.

Our data strongly support a direct regulatory interaction between Ern0160 and these mRNAs: both targets are downregulated upon Ern0160 overexpression (Fig. 4A), and electrophoretic mobility shift assays confirmed the formation of stable sRNA–mRNA complexes (Fig. 4C). Moreover, complex formation was abolished when a 13-nucleotide deletion was introduced in the base-pairing region of the target mRNAs (*01059/01150mut*) (Fig. 4D), or when the corresponding binding site was deleted from the sRNA itself (Ern0160mut13), reinforcing the specificity of this interaction (Fig. 4A).

**Fig. 6:**
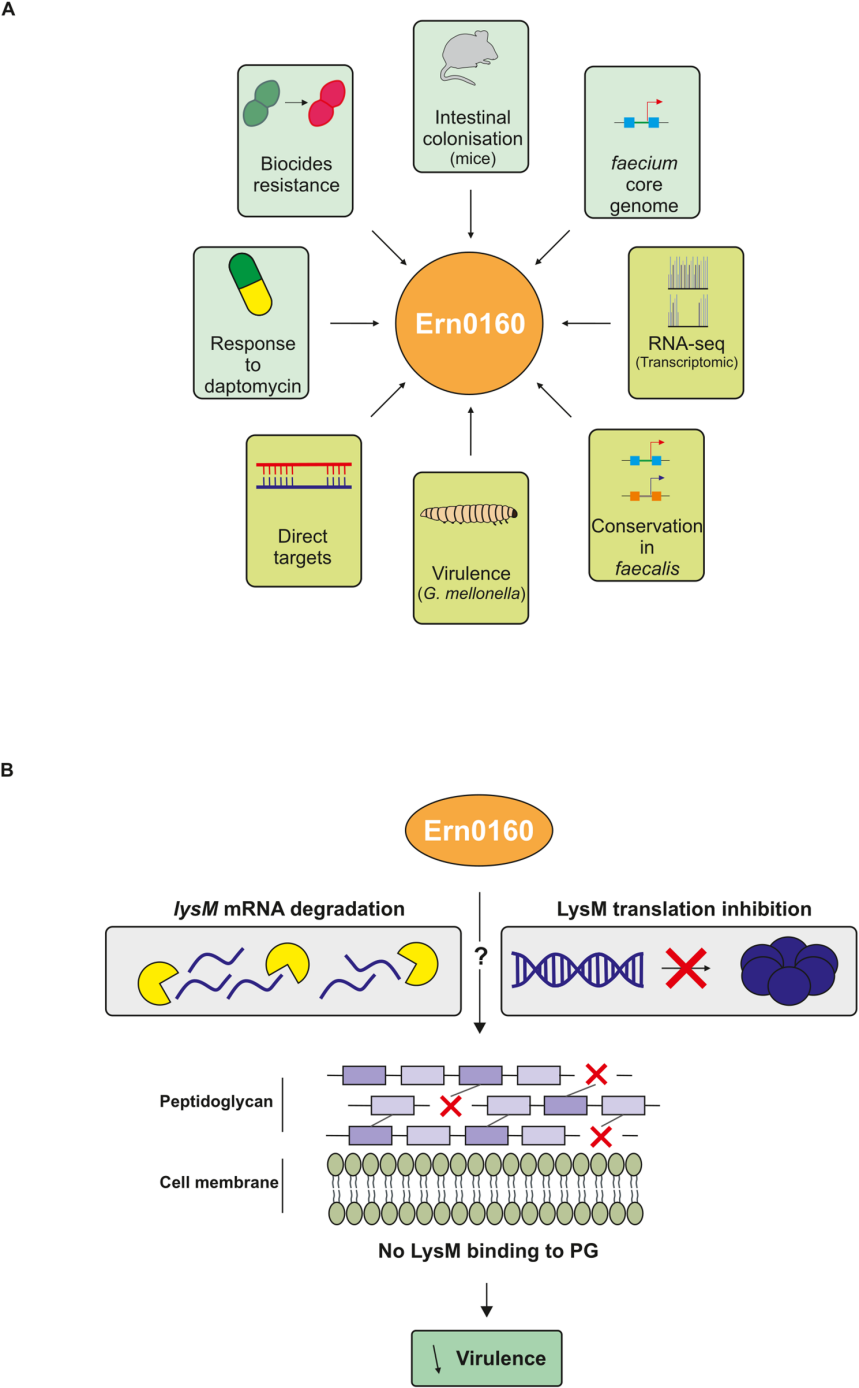
Proposed model. **A** Summary of known and newly uncovered roles of Ern0160. Previously reported functions are indicated in pastel green; novel findings from this study are shown in light green. **B** Proposed mechanism: Ern0160 represses *LysM* expression either through RNase-mediated mRNA degradation or by blocking ribosome binding, possibly via structural occlusion of the RBS. Although no RNA-binding protein (RBP) partners were identified, their involvement cannot be excluded. Reduced LysM protein levels would impair peptidoglycan binding and downstream virulence mechanisms

While the precise molecular mechanism of Ern0160-mediated repression remains to be elucidated, its location of binding—within the open reading frame rather than the 5′ UTR—raises intriguing possibilities (Fig. 6). Although many bacterial sRNAs block translation by occluding the ribosome binding site (RBS), often via 5′ UTR interactions [32], our findings suggest that Ern0160 may instead induce conformational changes that render the RBS inaccessible, or may even destabilize the transcript through recruitment of yet-unidentified RNase partners. Grad-seq data we previously published did not reveal any co-migrating RNA-binding proteins commonly associated with sRNA function, such as S1, cold-shock proteins, or Khp family members [29], indicating that Ern0160 may act independently or through novel interaction partners.

The biological significance of Ern0160 is further underscored by the functional roles of its targets. Both EFAU004_01059 and 01150 were previously identified as virulence factors in a mouse model of infection [20], being upregulated in bacteria isolated from host tissues. Deletion of both genes resulted in impaired colonization of kidneys and bladder. These genes encode proteins with LysM domains, a widespread structural motif involved in peptidoglycan binding, implicated in diverse bacterial processes including cell wall remodeling, immune evasion, and adhesion [27]. LysM-containing proteins from pathogens such as *S. aureus*, *Francisella tularensis*, *Neisseria meningitidis*, and *E. coli* have been directly linked to virulence and host cell interactions [33–36]. In *E. faecium*, the exact mechanism of *01059/01150*-mediated virulence is unknown, but it likely involves modulation of the cell wall to enhance fitness under host-imposed pressures.

Given this context, Ern0160 may act as a molecular switch that fine-tunes virulence gene expression depending on environmental conditions (Fig. 6). Previous in vivo studies have shown that Ern0160 expression enhances intestinal colonization, but excessive levels become detrimental [11], pointing to the need for tight regulatory control. Here, we extend these findings using the *Galleria mellonella* infection model and demonstrate that deletion of Ern0160 increases host mortality, whereas its overexpression reduces virulence. Notably, this attenuation was reversed when using the mutant Ern0160mut13, which is incapable of target repression (Fig. 5). These results support a model in which Ern0160 suppresses pro-virulent LysM gene expression, maintaining *E. faecium* in a commensal state even under conditions that would otherwise promote virulence. If we decided to focus on both LysM direct Ern0160 targets, our RNA-seq results under Ern0160 overexpression also revealed enrichment of different functional categories that could be explored in future studies (Fig. 2C and Table S4). Notably, the most significantly downregulated genes belong to categories linked with carbohydrate utilization and transport, whereas the most significantly upregulated genes are associated with amino acid transport and metabolism, as well as transcription and translation pathways. It is also worth noting that the expression of five sRNAs appears to be affected by Ern0160, particularly one of unknown function, Ern0170, which is highly upregulated. This observation may highlight cross-talk between multiple sRNAs in target regulation in *E. faecium*.

Furthermore, although proteomics was beyond the scope of the present study, a targeted or global proteomics approach—particularly focusing on cell-wall–associated proteins—would be an informative avenue for future investigations into Ern0160-dependent regulation.

To our knowledge, this is the first report to identify a direct sRNA-mRNA regulatory network in *E. faecium*. Our study not only uncovers Ern0160 as a key post-transcriptional regulator of virulence but also provides a framework for understanding how sRNAs contribute to niche adaptation in opportunistic pathogens.

### Limitation of the study

While our integrative approach provides converging evidence for a direct regulatory role of Ern0160 on its LysM targets, several limitations should be acknowledged. Although overexpression is widely used to reveal sRNA–mRNA interactions that may be silent under standard laboratory conditions, it does not fully reproduce the native expression levels or induction dynamics of Ern0160 and does not constitute a true genetic complementation of the deletion mutant. The virulence attenuation observed in the overexpression strain should therefore be interpreted with caution, as it may reflect non-physiological regulatory states rather than an in vivo response. Moreover, because sRNAs primarily act at the post-transcriptional level, the absence of protein-level measurements is an additional limitation, as mRNA abundance may not completely reflect regulatory output. These considerations frame the interpretation of our virulence findings and underscore the value of future work employing physiological expression systems and proteomic approaches to refine the biological role of Ern0160.

## Conclusion

In this study, we characterized the sRNA Ern0160 as a novel regulator of virulence in *Enterococcus faecium*. Through an integrated approach combining transcriptomics, in silico predictions, biochemical validation, and in vivo infection models, we identified and validated *EFAU004_01059* and *EFAU004_01150* as direct targets of Ern0160. These genes encode LysM domain-containing proteins previously implicated in virulence, and their expression is repressed by Ern0160 through a specific and direct RNA-RNA interaction.

Importantly, the functional consequences of Ern0160-mediated repression were evident in the *G. mellonella* infection model, where loss of Ern0160 increased virulence, while its overexpression—if retaining target binding capacity—attenuated infection. These findings demonstrate that Ern0160 acts as a fine-tuning regulator of virulence, likely helping *E. faecium* navigate the transition between commensalism and pathogenicity in response to environmental stimuli.

Our results highlight the power of sRNA-based regulation in bacterial adaptation and pathogenesis, and set the stage for future studies to explore Ern0160-associated pathways and regulatory networks. Targeting such sRNA-mediated mechanisms may represent a novel strategy to mitigate opportunistic infections caused by multidrug-resistant *E. faecium*.

## Supplementary Information

Supplementary Figure 1.

Supplementary Figure 2.

Supplementary Figure 3.

Supplementary Table 1.

Supplementary Table 2.

Supplementary Table 3.

Supplementary Table 4.

## Data Availability

All data generated or analysed during the current study are included in this published article and its supplementary information files. Raw and processed RNA-seq data have been deposited in the Gene Expression Omnibus (GEO) repository at the National Center for Biotechnology Information (NCBI) under accession number GSE239910.
