# Supplementary figures and images for: Regulatory RNA Ern0160 controls Enterococcus faecium virulence through direct modulation of expression of LysM domain-containing proteins
Source: BMC Genomics. 2026 Jan 5;27:126. doi: 10.1186/s12864-025-12464-2 (PMC12870237; doi:10.1186/s12864-025-12464-2)

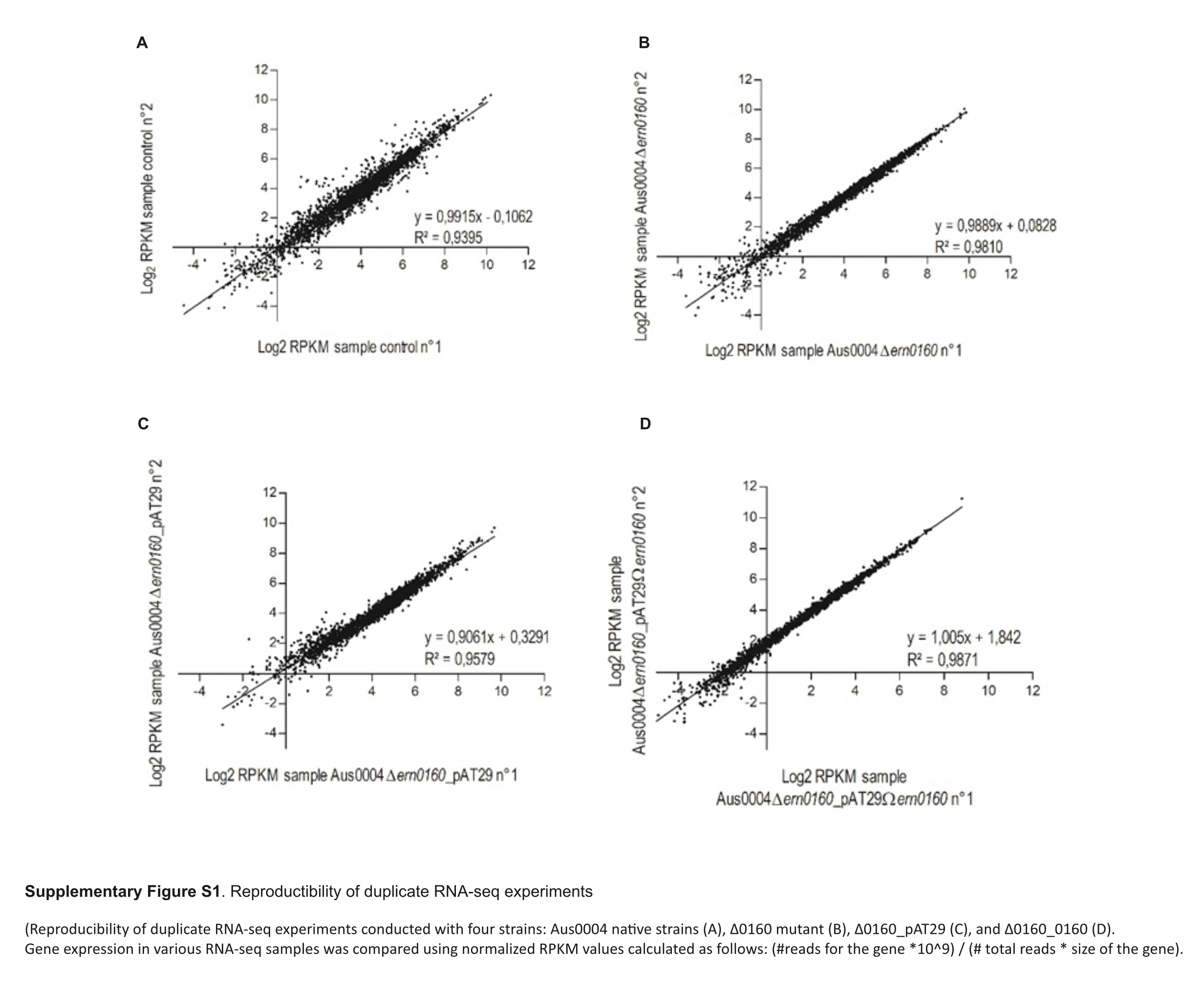

Supplement: Supplementary file 1 — Supplementary Figure 1. [file 12864_2025_12464_MOESM1_ESM.tif]

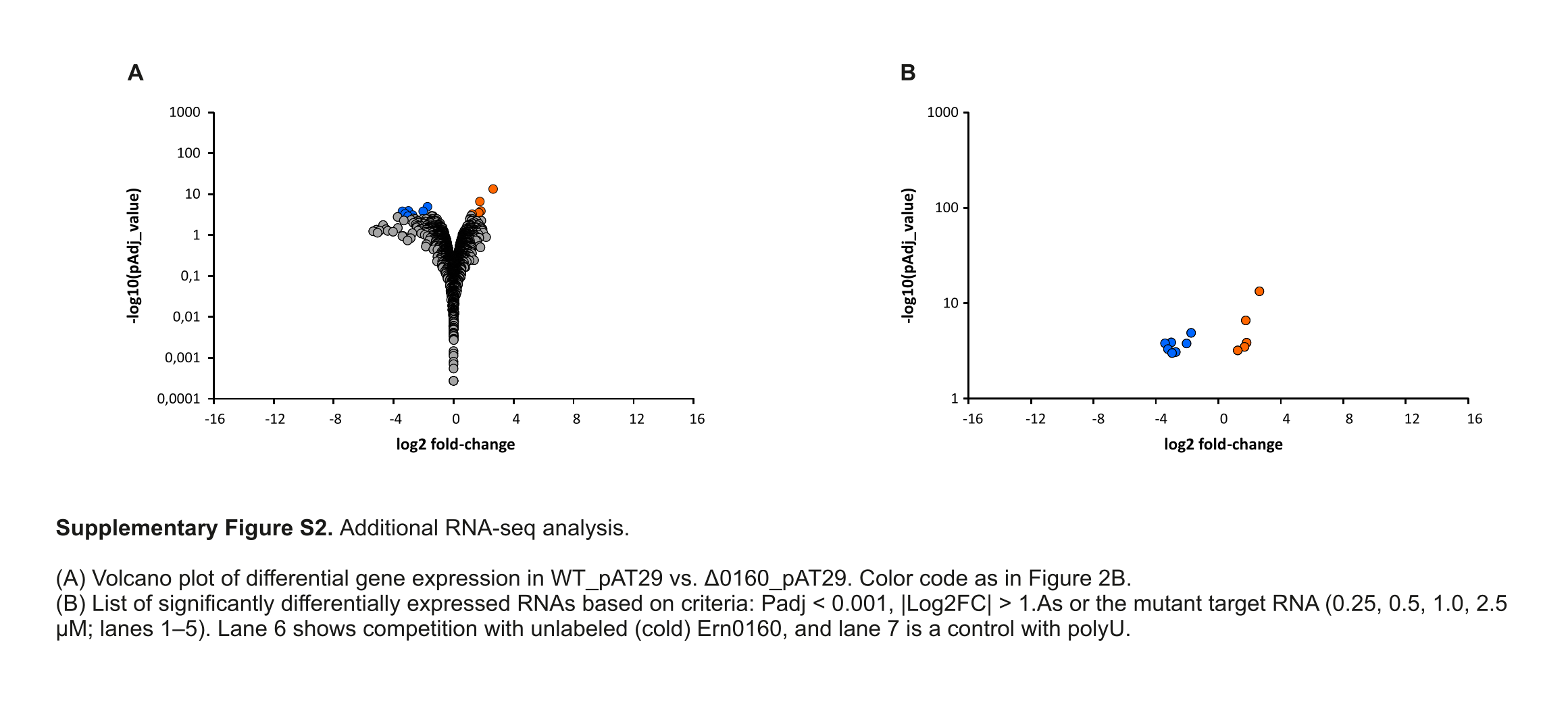

Supplement: Supplementary file 2 — Supplementary Figure 2. [file 12864_2025_12464_MOESM2_ESM.tif]

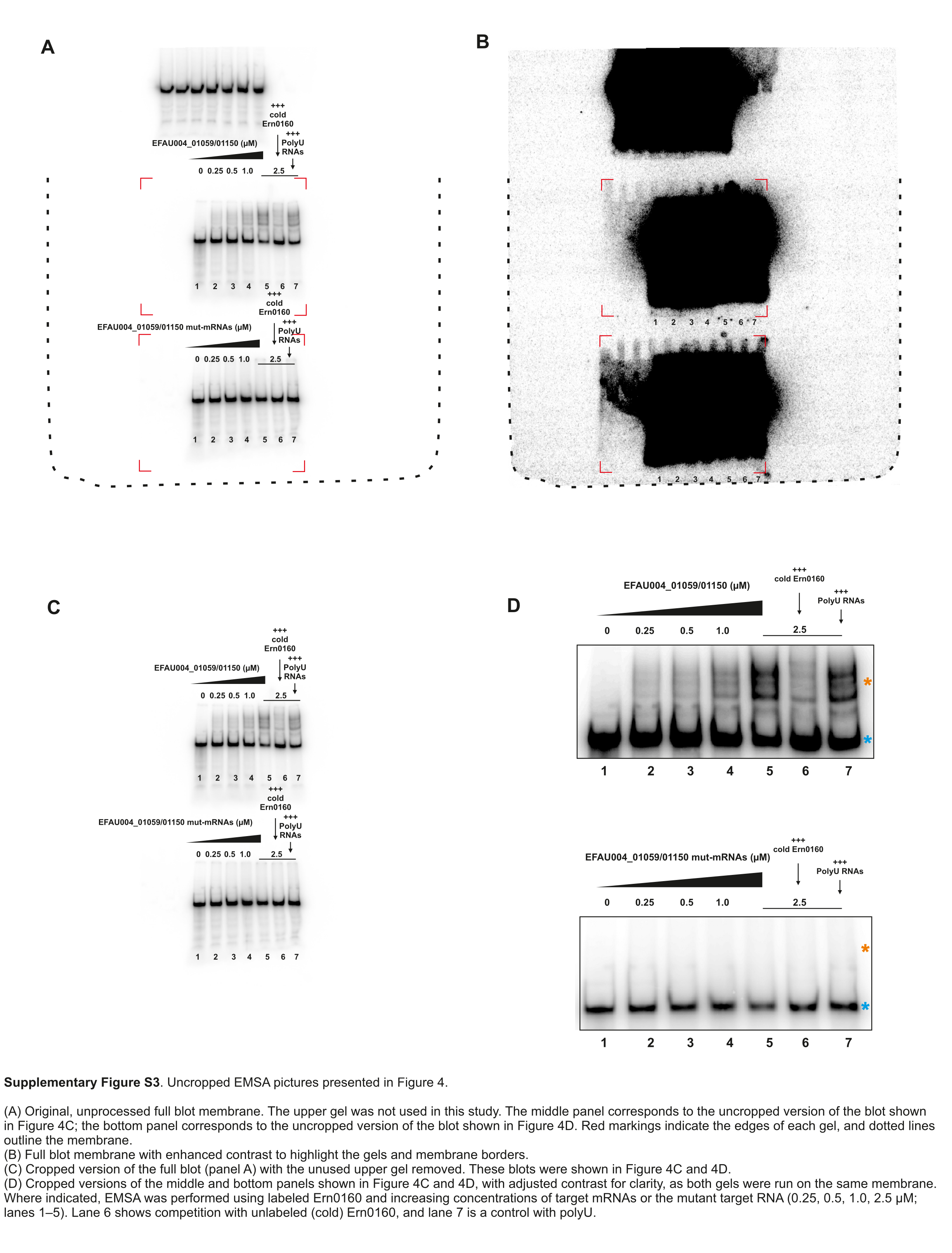

Supplement: Supplementary file 3 — Supplementary Figure 3. [file 12864_2025_12464_MOESM3_ESM.tif]
